# Clinical outcomes following surgical fixation of acromion fractures

**DOI:** 10.1016/j.jseint.2024.11.016

**Published:** 2024-12-04

**Authors:** Tram L. Tran, Molly G. Sekar, Nik Bhardwaja, Jessica McGraw-Heinrich, Michael D. McKee, Niloofar Dehghan

**Affiliations:** a Department of Orthopaedic Surgery, University of Arizona College of Medicine Phoenix, Phoenix, AZ, USA; b Department of Orthopaedic Surgery, The CORE Institutes, Phoenix, AZ, USA

**Keywords:** Acromion, Acromial, Scapula, Fracture, Reverse shoulder arthroplasty, Periprosthetic, Stress fracture, Surgical fixation

## Abstract

**Background:**

Acromial fractures are rare in the traumatic setting; however, they have recently gained attention due to the increase in incidence as a result of a postoperative complication of reverse total shoulder arthroplasty. While historically these fractures were routinely treated nonoperatively, there is evidence that surgery can improve outcomes. The study aims to evaluate clinical outcomes following surgical treatment of acromion fractures and compare outcomes among patients with an intact rotator cuff against those with a deficient rotator cuff or reverse shoulder arthroplasty.

**Methods:**

This is a retrospective review of patients with acromion fractures that were treated with open reduction internal fixation between January 2014 and March 2023. Patients were stratified into three cohorts as follows: 1) rotator cuff intact; 2) rotator cuff impaired; and 3) presence of reverse total shoulder arthroplasty.

**Results:**

Thirty-seven patients were included in the study with a mean follow-up of 9 months (range 0.5-77). The mechanism of injury was stress fracture (46%), high-energy trauma (32%), or low-energy falls (22%). The mean time to surgery was 6 months (0-24). Older age and female sex were associated with stress fractures (*P* < .05). The odds of having a stress fracture were higher in patients with an impaired rotator cuff (OR 6.5, *P* = .04) or reverse total shoulder arthroplasty (OR 2.8, *P* = .02) compared to those with an intact rotator cuff. The mean shoulder flexion improved from 81 degrees preoperatively to 113 degrees at the time of the last visit (*P* = .02). The mean shoulder external rotation improved from 24 degrees preoperatively to 48 degrees at the time of the last visit (*P* = .04). The nonunion rate was 19% (7 of 37) and the reoperation rate was 11% (4 of 37) for removal of broken hardware or nonunion revision. Two patients went on to have reverse total shoulder arthroplasty. There were no differences in nonunion or reoperation rates among patients with an intact cuff, an impaired cuff, or the presence of shoulder arthroplasty.

**Discussion and conclusion:**

Patients with rotator cuff dysfunction or presence of reverse total shoulder arthroplasty are more likely to have acromion stress fractures compared to those with an intact rotator cuff. Surgical fixation of acromion fractures can improve the shoulder range of motion and pain scores. The nonunion rate is lower for surgical fixation compared to existing literature on nonoperative treatment of acromion fractures.

Acromial fractures are rare in the traumatic setting; however, they have recently gained attention due to the increase in incidence as a result of a postoperative complication of reverse total shoulder arthroplasty (rTSA). The mechanism for traumatic fractures is often a result of a direct blow and is often associated with other shoulder girdle injuries.^1^ However, the mechanism for fracture in the setting of rTSA is often a stress fracture due to the longer arm length and increase in abduction moment and force on the deltoid muscle origin.^2^ Most recently, it has been hypothesized that a shoulder with a dysfunctional or absent rotator cuff may impose similar biomechanics and can be at risk for developing acromial stress fractures.^3^^,^^4^

There is a paucity of literature evaluating clinical outcomes for conservative or surgical treatment and management of acromion fractures. In the setting of rTSA, multiple studies report inferior clinical outcomes in patients with nonoperatively treated acromion fractures, compared to those without acromion fractures.^5^ One study reported higher union rates with operative treatment compared to nonoperative treatment, but there was no difference in shoulder range of motion or patient outcomes.^6^ Displaced fractures or nonunion can lead to poor shoulder function and chronic pain.^7^^,^^8^ Studies reporting clinical outcomes following operative treatment for acromion fractures are limited to small case series. The primary aim of this study is to evaluate clinical outcomes following surgical treatment of acromion fractures. In our practice we have noticed that acromial fractures are more common in cuff-deficient shoulders, such as patients with large rotator cuff tears or rTSA for rotator cuff arthropathy. Specifically, this study aims to compare outcomes among patients with an intact rotator cuff against those with a deficient rotator cuff or reverse shoulder arthroplasty.

## Materials and methods

After obtaining institutional review board approval, a retrospective chart review was performed on all patients who underwent surgical fixation of an acromion fracture between January 2014 and March 2023. Patients who had undergone surgical fixation of scapula fractures were identified using the Current Procedure Terminology code 23585 (open treatment of scapula fracture). The inclusion criteria were isolated acromion fractures treated with open reduction internal fixation and age over 18. Patients undergoing surgical treatment for an os acromiale or fixation of the acromion as part of a traumatic scapula or glenoid fracture were excluded. Medical chart review was conducted to collect demographic data, including age, gender, smoking status, and comorbidities, as well as mechanism of injury, duration of symptoms, preoperative and postoperative range of motion, postoperative weight-bearing status, and patient-reported pain scores. Patients were divided into three cohorts as follows: 1) rotator cuff intact; 2) rotator cuff impaired; and 3) the presence of rTSA. Rotator cuff pathology was defined by a documented medical history of rotator cuff symptoms or prior rotator cuff repair. Furthermore, advanced imaging (either computed tomographyscan or magnetc resonance imaging) was reviewed to assess the structural integrity of the rotator cuff. Patients with no prior history or symptoms of rotator cuff disease were assumed to have an intact rotator cuff. Surgical indications were determined by the treating surgeon. In the cases of nonunion or stress fractures, surgical indications were continued pain and shoulder dysfunction after failure of conservative treatment.

Statistical analyses were performed using StataCorp. 2021(Stata Statistical Software: Release 17; StataCorp, College Station, TX, USA). Descriptive statistics include frequencies and proportions for categorical variables, and mean, standard deviation (SD), and five-number summaries for continuous variables. Fisher’s exact test was used to compare categorical data and Student *t*-*test* for continuous data. Univariate and multivariate logistic regression was used to evaluate predictors of the outcome. The statistical significance level is 0.05.

## Results

### Patient and fracture characteristics

Thirty-seven patients were included in the study with a mean follow-up of nine months (range 0.5-77). The mean age was 64 years (range 25-88), and 35% were female. 16% (6/37) had diabetes, and 51% (19/37) were never smokers. The mechanism of injury was categorized as stress fracture or chronic nonunion (16/37, 43%), high-energy trauma (13/37, 35%), or low-energy injury or ground-level falls (8/37, 22%). 16 patients had stress fracture or nonunion as the initial mechanism, with a mean age of 69.9 years (±7.4). The majority of these patients had rotator cuff deficiency as follows: intact cuff n = 3, impaired cuff n = 6, and rTSA n = 7.

**Figure 1:**
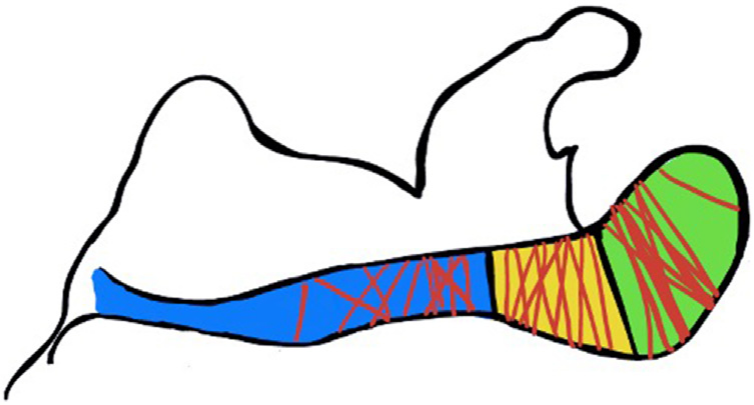
Heat map of the fractures in the study cohort based on Levy classification.

The mean preoperative shoulder flexion was 80 degrees (range 15-160), and the mean preoperative shoulder external rotation was 24 degrees (range 0-50). Patient demographics were similar among the three groups (Table I). Fractures were classified according to the Levy classification^9^ (Fig. 1).

**Table I:** Study cohort characteristics by rotator cuff status vs. rTSA.

Characteristics	Intact cuff (n = 16)	Cuff impaired (n = 10)	Reverse arthroplasty (n = 11)	*P* value
Age, y	58 (18)	67 (10)	70 (6)	.08
Female sex	3 (23%)	3 (23%)	7 (54%)	.06
Smoking status				.31
Never	9 (47%)	6 (32%)	4 (21%)	
Past	4 (67%)	0 (0%)	2 (33%)	
Current	3 (25%)	4 (33%)	5 (42%)	
Diabetes	3 (50%)	3 (50%)	0 (0%)	.17
Mechanism				<.001
Stress or chronic nonunion	3 (18%)	6 (38%)	7 (44%)	
High energy	12 (92%)	1 (8%)	0 (0%)	
Low energy	1 (12%)	3 (38%)	4 (50%)	
Length of follow up, mo	5 (5)	12 (17)	13 (22)	.42
Duration of symptoms prior to surgery, months	3 (4)	9 (9)	7 (8)	.09
Preoperative range of motion, degrees				
Shoulder flexion	91 (54)	65 (54)	83 (38)	.61
Shoulder external rotation	38 (8)	15 (7)	20 (19)	.24

Categorical data are presented as number (percentage) and continuous data are presented as mean (standard deviation).

### Surgical details

The mean time from injury to surgery was six months (range 0-24). The mean time to surgery from initial evaluation for patients with stress fractures or nonunion was 18.1 week (±18.3), compared to 3.3 weeks (±6.6) for acute fractures. The most common construct used for surgical fixation was two 2.7 mm compression plates (57%), followed by one compression plate (35%) and other constructs, such as screws, suture, or wire (8%). Local bone graft or allograft was used in 39% of the cases (14 of 37). Three cases underwent concomitant rTSA at the time of acromion fixation. Seven cases were acromion nonunions as follows: four treated with a single-plate fixation and three with dual-plate fixation. There were no associations found between the type of implant or bone graft usage and nonunion or reoperation rates.

### Shoulder range of motion after surgery

**Figure 2:**
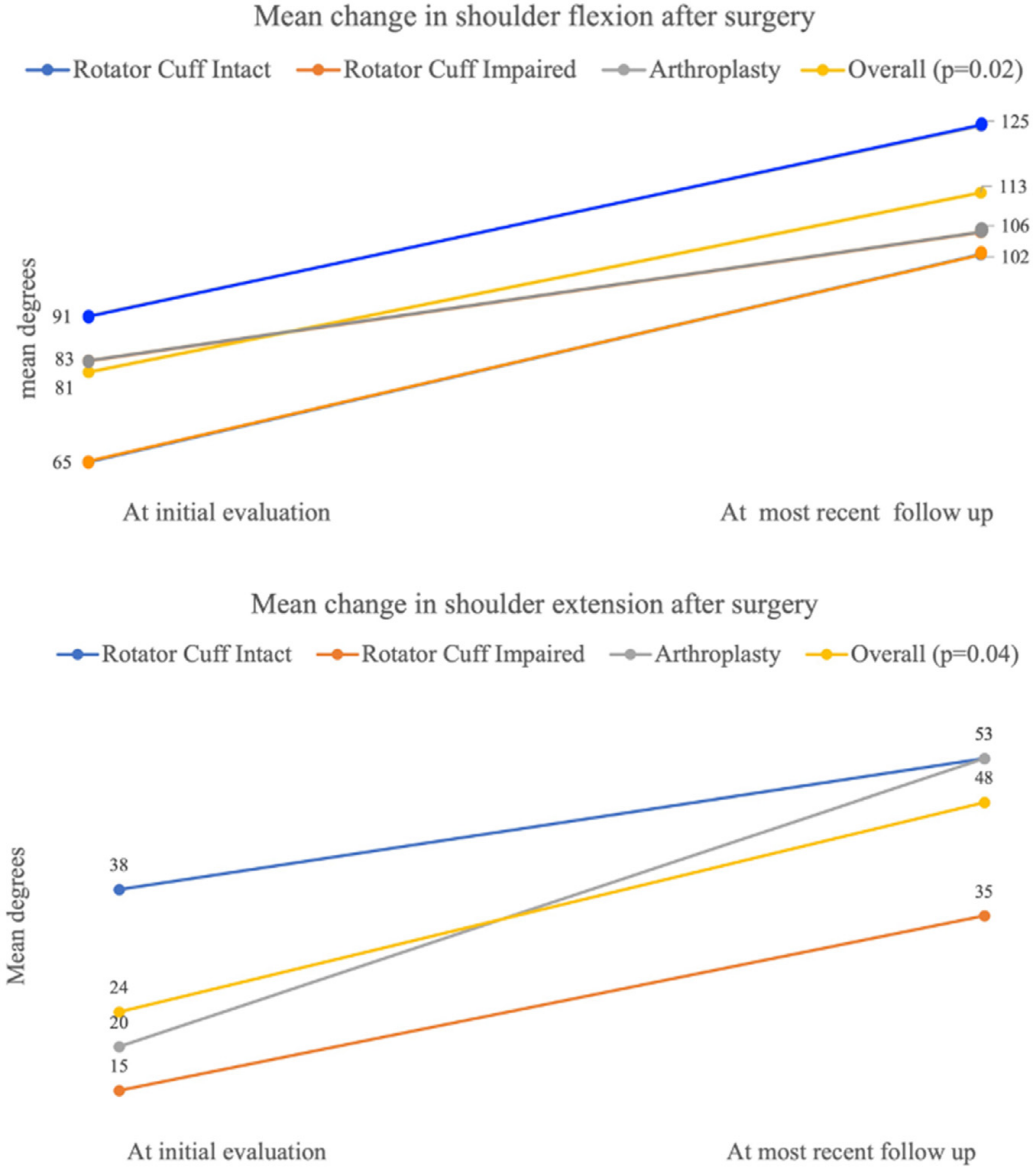
Preoperative and postoperative shoulder range of motion among rotator cuff intact vs. rotator cuff impaired vs. reverse arthroplasty.

94% of patients were made nonweight-bearing initially after surgery. Patients were allowed weight-bearing as tolerated at a mean of 35 days (range 7-80) postoperatively. At the most recent follow-up visit, the mean pain score was 3 out of 10 (range 0-7). Among all patients, the mean shoulder flexion improved from 81 degrees (SD 47) preoperatively to 113 degrees (SD 9) at the time of the last visit (*P* = .02), and the mean shoulder external rotation improved from 24 degrees (SD 5) preoperatively to 48 degrees (SD 12) at the time of the last visit (*P* = .04) (Fig. 2). Although shoulder range of motion improved among the entire cohort, there were no observed differences among those with intact rotator cuff, impaired rotator cuff, and the presence of arthroplasty (Table II). Three patients were lost to follow-up.

**Table II:** Postoperative range of motion and outcomes based on cuff status.

Outcome	Intact cuff	Cuff impaired	Reverse arthroplasty	*P* value
Mean shoulder flexion improvement following surgery (degrees)				.02
At the initial visit	38 (53)	65 (54)	83 (38)	
At the last clinic visit	125 (44)	102 (23)	106 (38)	
Mean shoulder external rotation improvement following surgery (degrees)				.04
At the initial visit	38 (8)	15 (7)	20 (19)	
At the last clinic visit	53 (11)	35 (7)	53 (51)	
Pain score (n = 17)	2 (2)	5 (3)	3 (2)	.3
Nonunion				.6
Stress or chronic fractures	0 of 16 (0)	2 of 10 (20)	2 of 11 (18)	
Acute fractures	2 of 16 (13)	0 of 10 (0)	1 of 11 (9)	
Reoperation	1 of 16 (6)	3 of 10 (30)	1 of 11 (9)	.3

Data are presented as number (column percentage) and continuous data are presented as mean (standard deviation).

### Nonunion and reoperation rates

The nonunion rate was 19% (7 of 37) overall. Among those, 72% (5 of 7) were from acute fractures and 28% (2 of 7) were from chronic stress fractures. The mean age for patients who went on to a nonunion was 72 years, and 4/7 were female. Fracture locations were two Levy type 1, two Levy type 2, and three Levy type 3. The mean time to surgery among those who went on to have a nonunion was 9 months compared to 5 months among those whose fracture healed (*P* = .1). There were no statistically significant associations between the nonunion rates and mode of fixation, time to surgery, age, female sex, smoking status, or Levy classification. Five of the seven patients with nonunion were treated conservatively and two patients went on to have revision fixation (one at five months and one at two years after initial surgery). The reoperation rate was 11% (4 of 37) for removal of broken hardware or nonunion revision. Two patients went on to have rTSA. Postoperative shoulder motion, nonunion, and reoperation rates are similar among those with an intact rotator cuff, impaired rotator cuff, and arthroplasty (Table II). Postoperative shoulder motion, nonunion, and reoperation rates are similar among fracture location based on Levy classification (Table III).

**Table III:** Postoperative range of motion and outcomes based on Levy Classification.

	Levy 1 (n = 13)	Levy 2 (n = 12)	Levy 3 (n = 12)	*P* value
Nonunion	2 (15)	2 (17)	3 (25)	.9
Reoperation	2 (15)	0 (0)	3 (25)	.3
Shoulder flexion (n = 23), degrees	103 (34)	130 (42)	90 (8)	.9
Shoulder external rotation (n = 7), degrees	40 (0)	49 (34)	∗	.9

Data are presented as number (column percentage) and continuous data are presented as mean (standard deviation).

∗ There were no available data for this category.

### Predictors of acromion stress fractures

A univariate analysis showed that older age (OR 1.0, 1.0-1.1 95% CI, *P* = .04) and female sex (OR 5.5 1.3-23.8 95% CI, *P* = .02) was associated with stress fracture or chronic nonunion (Table IV). The odds of having a stress fracture or chronic nonunion is higher in patients with an impaired rotator cuff (OR 6.5, 1.1-38.6 CI, *P* = .04) or rTSA (OR 2.8, 1.1-6.6 CI, *P* = .02) compared to those with an intact cuff. In a multivariate analysis adjusting for older age and female sex, impaired rotator cuff or presence of arthroplasty was no longer a significant predictor for stress fractures (Table IV). Location of fracture based on Levy classification was not a significant predictor of stress fractures.

**Table IV:** Univariable and multivariable logistic regression analysis of acromial stress fractures.

	Univariable			Multivariable		
	OR	95% CI	*P* value	OR	95% CI	*P* value
Female sex	5.5	1.3-23.8	.02	34.1	0.7-23.7	.1
Age (y)	1.0	1.0-1.1	.04	1.0	1.0-1.1	.3
Levy classification						
Levy 1 (reference)	-	-	-	-	-	-
Levy 2	2.3	0.4-11.5	.3	2.0	0.3-15.4	.1
Levy 3	2.3	0.4-11.5	.3	0.9	0.1-6.8	.3
Rotator cuff status						
Intact cuff (reference)	-	-	-	-	-	-
Cuff impaired	6.5	1.1-38.6	.04	7.5	1.0-59.1	.1
Reverse arthroplasty	2.8	1.1-6.6	.02	2.0	0.7-5.5	.1

*OR*, odds ratio; *CI*, confidence interval.

## Discussion

Acromial and scapular spine fractures can occur as a result of acute trauma, secondary to chronic stress reactions, or as a complication secondary to rTSA. Literature on these injuries is limited and outcomes after surgical fixation remain to be further studied. This study reports clinical outcomes following surgical fixation of acromial fractures in 37 patients. To our knowledge, this is the largest cohort study to date reporting functional outcomes after surgical treatment of these injuries.

Isolated traumatic acromion fractures are rare, as the acromion is often injured in conjunction with other scapular or shoulder girdle injuries. Indications for surgical treatment are not well-established. Previous studies on these injuries are limited to small case series and while variation exists in treatment recommendations and surgical fixation techniques, many authors recommend anatomic reduction and fixation to prevent symptomatic nonunion.^1^^,^^8^ In our series, 13 of the 37 acromion fractures were secondary to high-energy trauma. Surgical treatment of these fractures with plate and screw constructs resulted in reliable union, with 12 of 13 fractures uniting. Our results are similar to those noted by previous authors’ case series.^1^^,^^8^

Acromion fractures occurring secondary to chronic stress reactions or as a complication secondary to rTSA are being increasingly studied with exponentially increasing rates of rTSA procedures. The two largest published series evaluating surgical treatment of acromial fractures secondary to these mechanisms evaluated 7 and 11 patients.^6^^,^^10^ These studies note a nonunion rate in operatively treated fractures of 41% and 18%, respectively. In our cohort, operative treatment of chronic nonunion and/or stress fractures resulted in 12 of 16 fractures healing, with a 25% rate of nonunion (4/16). In contrast, prior studies have noted much higher nonunion rates in non-operatively treated fractures, ranging from 41% to 61.4%.^5^^,^^6^^,^^10^

There are two studies that compare conservative vs. operative treatment for acromion fractures. One series of 7 operative and 16 nonoperative fractures found no difference in Constant-Murley score, range of motion, and subjective shoulder value among patients treated with conservative or operative treatment.^6^ Another series of 11 operative and 35 nonoperative fractures showed similar clinical outcomes between groups for shoulder range of motion, abduction strength, subjective shoulder value, and Constant–Murley scores.^10^ The null difference in findings of these studies is likely attributable to a small sample size. Our series showed improvements in shoulder range of motion after operative treatment compared to preoperative measurements, which suggests that there is a subset of patient who fail conservative treatment and would benefit from operative fixation.

Risk factors for acromial stress fractures following rTSA have been increasingly evaluated. Multiple studies have shown that female sex, rheumatoid arthritis, osteoporosis, increased length and tension of the deltoid, and increased glenohumeral center-of-rotation medialization are associated with stress fractures.^3^^,^^4^^,^^11^ It is hypothesized that altered shoulder biomechanics from rTSA that put the deltoid at a mechanical disadvantage subsequently put increased stress on the deltoid origin. In patients with osteoporosis or decreased bone density, this increased stress can result in acromial stress fractures.^11^

Our study has multiple limitations. The retrospective nature of our series carries potential for selection bias inherent to such studies. While this is the largest case series to date, our series still has a relatively small sample size in which we only evaluate operatively treated acromion fractures. The rotator cuff assessment was conducted clinically as documented in the medical record and confirmed with computed tomographyscan assessment. However, given the lack of magnetic resonance imaging, we cannot confirm the structural status of the rotator cuff. No assumptions can be made in how our outcomes compare to nonoperatively treated fractures, as these were not evaluated in our series. Lastly, our group of patients is heterogenous owing to varying fracture mechanisms and variability in the presence or absence of rTSA components. Ongoing studies are needed to further evaluate treatment methods, surgical indications, and optimal fixation constructs.

## Conclusions

Surgical fixation of acromion fractures can improve shoulder range of motion and pain scores. The nonunion rate is lower for surgical fixation compared to existing literature on conservative treatment of acromion fractures. Clinical improvement cannot be compared to conservative treatment and would necessitate further studies.

## Disclaimers:

Funding: No funding was disclosed by the authors.

Conflicts of interest: The authors, their immediate families, and any research foundation with which they are affiliated have not received any financial payments or other benefits from any commercial entity related to the subject of this article.
